# Use of encapsulated dexamethasone sodium phosphate (eDSP) in chronic obstructive pulmonary disease, cystic fibrosis, and inflammatory bowel disorders

**DOI:** 10.3389/fddev.2025.1730142

**Published:** 2025-12-08

**Authors:** Biljana Horn, Giovanni Mambrini, Maureen Roden, Caralee Schaefer, Dirk Thye, Mauro Magnani

**Affiliations:** 1 Quince Therapeutics, Inc., South San Francisco, CA, United States; 2 Department of Biomolecular Sciences, University of Urbino, Urbino, Italy

**Keywords:** erythrocyte encapsulated dexamethasone sodium phosphate (eDSP), EryDex, inflammatory bowel disease, cystic fibrosis, chronic obstructive pulmonary disease, osmotic encapsulation

## Abstract

Glucocorticoids are cornerstone treatment for inflammatory diseases but are limited by systemic toxicity from high-dose and prolonged use. Encapsulation of dexamethasone sodium phosphate (DSP) in autologous erythrocytes aims for sustained drug release with an improved safety profile. This manuscript summarizes early clinical studies of encapsulated DSP (eDSP) in pulmonary and inflammatory bowel disorders (IBD). From 2001 to 2013, eight clinical studies investigated eDSP in patients whose age ranged from 5 to 83 years, with chronic obstructive pulmonary disease (COPD), cystic fibrosis (CF), Crohn’s disease (CD), and ulcerative colitis (UC). DSP was loaded into autologous erythrocytes *ex vivo* and reinfused every 2 weeks, or monthly. Follow-up ranged from 1 to 24 months. In pulmonary indications, eDSP resulted in improved FEV1 and reduced infections in CF patients, and improved symptoms in COPD with markedly reduced corticosteroid doses. In IBD, eDSP enabled steroid withdrawal in 60%–78% of patients and achieved remission in pediatric and adult CD and UC. Adverse effects typical of corticosteroids were notably absent. Limitations of these studies included small sample sizes, lack of placebo groups in some trials, and inter-patient variability in erythrocyte drug loading. Pharmacokinetic studies documented persistence of dexamethasone levels up to 4 weeks post-infusion. Early studies demonstrate that eDSP is a feasible and well-tolerated treatment in children and older patients, delivering low-dose corticosteroids with prolonged therapeutic levels. These findings support further development of erythrocyte-based drug delivery for chronic inflammatory diseases in patients with steroid sensitive or steroid-dependent disease.

## 1 Introduction: delivering corticosteroids via red blood cells

Corticosteroids remain an important treatment for inflammatory diseases (Baschant and Tuckermann, 2010; Strehl et al., 2019) but are limited by side effects (Aljebab et al., 2017). Due to the short half-life of synthetic corticosteroids, high daily doses are required to achieve a therapeutic effect. High daily doses, used for longer than 2 weeks, invariably result in toxicities involving almost every organ system in a body (Schacke et al., 2002; Aljebab et al., 2017).

Alternative drug delivery systems for corticosteroids and other agents are under development. Their main goal is to maintain therapeutic benefit while minimizing toxicity. Red blood cells (RBCs) are emerging as one of the leading natural platforms for advanced drug delivery (Koleva et al., 2020; Berikkhanova et al., 2024; Zhang et al., 2024). Compared to synthetic carriers like liposomes or nanoparticles, RBC are biodegradable, can extend drug half-life, and deliver the drug in a sustained or targeted fashion (Berikkhanova et al., 2024). Two primary RBC drug delivery strategies are surface loading and encapsulation.

Different surface loading techniques have been studied preclinically. “RBC hitchhiking” combines nanomedicine and cell therapy. In mouse experiments, nanocarriers adsorbed to the RBC surface were transferred to the first organ downstream resulting in a ∼40-fold increased uptake compared to free nanocarriers (Brenner et al., 2018). An alternative approach comprised engineered single-chain antibody fragments (scFv) directed to human RBCs and fused with human thrombomodulin as a biotherapeutic cargo. Thrombomodulin fused to the RBC surface retained its function without compromising RBC deformability or response to osmotic stress (Villa et al., 2018). A recent review summarized different approaches for loading molecules to the RBC surface and for intracellular encapsulation (Glassman et al., 2020).

Osmotic encapsulation of drugs into RBC is a relatively simple and cost-effective technique. It is amenable to automation, paving the way for scalable manufacturing of cell-based therapeutics. Two drugs encapsulated into RBCs have advanced to phase 3 clinical trials.

Erycaps® a hypotonic-dialysis-based encapsulation was used to load L-asparaginase into homologous RBCs. Eryaspase (erythrocyte encapsulated L-asparaginase) clinical development is ongoing in lymphoid malignancies. It was well tolerated in patients with acute lymphoblastic leukemia. However, 9% of patients developed an allergic reaction accompanied by enzyme inactivation, since eryaspase was administered after hypersensitivity to PEG-asparaginase (Lynggaard et al., 2022). A large phase 3 study of eryaspase in combination with chemotherapy, versus chemotherapy alone, in patients with advanced pancreatic adenocarcinoma (Trybeca-1) did not meet primary endpoint in this difficult-to-treat population, and further development for this indication was abandoned (Hammel et al., 2025).

### 1.1 The EryDex system (EDS)

To reduce toxicity of dexamethasone, related to its short half-life requiring frequent administration and resulting in high peak levels after each dose, DSP was encapsulated in RBC using EDS to extend its intravascular half-life. EDS is classified by Food and Drug Administration (FDA) as a drug/device combination product and consists of a multi-use Red Cell Loader (RCL); a single-use EryKit_01, and a Syringe Kit; three processing solutions (Hypotonic solutions 1 and 2, and a hypertonic solution); and a drug DSP. The RCL system automates the 18-step process. Briefly, 50 mL of whole blood is obtained during each treatment visit from the patient and loaded into the RCL, hypotonic saline solutions are added to permit temporary reduction of osmolarity allowing diffusion of DSP into the cell. Subsequent addition of a hypertonic solution restores physiological osmolarity levels and entraps DSP within the RBC. The drug-loaded RBCs are washed automatically by the RCL to remove products of cell lysis, processing solutions, and non-encapsulated DSP. Sterility cultures are obtained, and the product is infused to the same participant over 30–50 min. *In vivo*, DSP slowly dephosphorylates (via enzymatic action within the RBC) to dexamethasone, which diffuses through the RBC membrane into the plasma. This delivers dexamethasone at stable, low levels over an extended period (Mambrini et al., 2017) This automated osmotic encapsulation was developed to address loading variability, noted in early clinical trials. The effect of encapsulation on the physiology of processed RBC was also described (Mambrini et al., 2017). A subsequent study assessed the pharmacokinetic (PK) properties of two dose levels (2.5–5 mg and 15–20 mg) of eDSP, given as a single infusion in healthy volunteers. Release of dexamethasone peaked 1 h after the end of IV infusion and dexamethasone was detected for 14 and 35 days after the 2.5–5 mg and 15–20 mg dose, respectively. The life span of DSP loaded RBC and controls, examined after radiolabeling with 51-Cr, was 84.3 ± 8.3 and 88.9 ± 6.2 days. *In vivo* recovery for DSP loaded RBC was similar to the 24-h recovery of RBC products intended for transfusion (Coker et al., 2018).

### 1.2 Clinical applications of eDSP

The safety and efficacy of eDSP has been studied in patients with ataxia telangiectasia in one phase 2 study (IEDAT-Ery01-2010), two phase 3 studies (IEDAT-02-2015 and IEDAT-04-2022), and their open-label extensions, with an aim at supporting potential regulatory submission in this rare disorder. Results from completed studies using eDSP in ataxia telangiectasia were published (Chessa et al., 2014; Leuzzi et al., 2015; Koenig et al., 2024; Zielen et al., 2024). eDSP was safe in this population and its use for 2 years or longer did not result in typical side effects seen with chronic corticosteroid use (Koenig et al., 2024). The confirmation of efficacy of eDSP in ataxia telangiectasia is expected upon completion of ongoing IEDAT-04-2022 trial.

The scope of this mini-review is to revisit early investigator initiated foundational clinical trials using eDSP (a.k.a. dex-21-P or EryDex) in pulmonary and inflammatory bowel disorders.

To include all trials PubMed searches were performed using the terms: “red blood cell loading,” “dexamethasone loading of erythrocytes,” “red blood cell encapsulation,” and “EryDex” and filtered to include only clinical studies for pulmonary and inflammatory disease. We also carefully reviewed recent reviews on erythrocyte-based drug delivery to ensure that relevant clinical systems in development were captured. Between 2001 and 2013, eDSP was used in 8 clinical trials, which are included in this mini-review We discuss the key findings on safety, transition from standard corticosteroids to eDSP, preliminary efficacy, and encapsulation variability and highlight contribution of early trials to the refinement of the EDS.

## 2 eDSP in chronic obstructive pulmonary disease

A summary of results from the three pulmonary studies is presented in Table 1.

**TABLE 1:** Summary of eDSP studies in Chronic Obstructive Pulmonary Disease and Cystic Fibrosis.

Study parameter	Rossi et al. (2001) Chronic Obstructive Pulmonary Disease, First Clinical Trial	Rossi et al. (2004) Cystic Fibrosis Phase 1/2 trial	Lucidi et al. (2006) Cystic Fibrosis – open label follow-up of Rossi’s study
Indication	COPD, advanced disease	CF with FEV1 ≥35%	CF with FEV1 <70%, pancreatic insufficiency and chronic pulmonary infections
Treatment groups	10 patients Patients 1-5 dose escalation group, 6-8 two doses of eDSP; 9-10 single dose followed by PK, patient 6 also had PK after the 4.4 mg dose	17 patients Patients 1-9 (PK and loading) Patients 9-17 (efficacy group) 9 contemporary controls	Out of 17 patients in Rossi's study 9 completed 24 months of treatment 9 contemporary controls
Patient age	Mean age 69 years Range 45-83 years	Age range 12-26 years, mean age of controls 18.8 years	Mean age 19.8 years Range 15-26 years
Dose of eDSP	0.8-8.8 mg in dose escalation group 3.5-6.5 mg in treated group 2.0 and 2.8 mg in PK group	1.19-14.5 mg in the PK group 8.9 ± 3.8 mg in the efficacy group	Not reported
PK findings	Plasma dexamethasone detectable for 7 days post infusion	Plasma dexamethasone detectable for 28 days post infusion. Dose was increased by adjusting erythrocyte incubation time during encapsulation (5-30 min)	Plasma dexamethasone levels of 0.1 to 0.2 nmol/ml documented up to one month after treatment
Frequency of treatment	Single dose (7 patients) Every 2 weeks x 2 treatments (3 patients)	Every 2 weeks x 16 treatments over 15 months	Monthly for 24 months
Outcome measures	Symptom diaries (breathing, cough) drug use cessation, physician evaluation of bronchospasm	FEV1, infection rate, use of antibiotics, IL-8 and TNF-α, BMI, BMD	FEV1 (z-score), Shwachman and Crispin indices, infection rates, safety including BMD, glucose and blood pressure
Time of assessment	1 month after the last eDSP dose	15 months after the start of treatment	24 months after the start of treatment
Treatment outcomes	Improved symptoms and physician assessed signs, decreased β-agonists and corticosteroids which were discontinued for 4-30 days	Increased mean FEV1 from 57% to 61% while in controls FEV1 decreased from 60% to 55%, 51% reduction in antibiotic cycles, no change in BMI, no change in BMD	Increased FEV1 (+1 standard deviation from baseline while control decreased 1.5 standard deviations from baseline), no change in clinical and nutritional status, similar infection rates
Steroid-related adverse events	None reported	None over 15 months	No evidence of diabetes, cataract, striae, hypertension or Cushingoid signs over 24 months of treatment
Main findings of the study	First clinical documentation of sustained release of dexamethasone from erythrocytes	First study in children showed no change in BMI or BMD after 15 months of therapy with eDSP	Safety after prolonged (24 months) treatment with eDSP was confirmed. eDSP improved FEV1 in this population

BMI, body mass index; BMD, bone mineral density; COPD, chronic obstructive pulmonary disease; CF, cystic fibrosis; eDSP, encapsulated dexamethasone sodium phosphate; FEV1, forced expiratory volume in 1 second; PK, pharmacokinetics; IL-8, interleukin 8; TNF-α, tumor necrosis factor alpha.

In Rossi et al., 2001 study, ten patients (45–83 years old) with COPD received eDSP. Five patients received a single administration with doses ranging from 0.78 to 8.78 mg; three patients received two infusions at 15-day intervals. A total of three patients had PK evaluation. Plasma dexamethasone levels remained detectable for up to 7 days post-infusion in patients receiving 2.00, 2.84, and 4.39 mg of eDSP. All patients showed reduced symptoms and discontinued β2 agonists and corticosteroids for durations ranging from 4 to 30 days.

This first clinical study demonstrated sustained release of dexamethasone from RBC, delivering approximately 25–50 times lower doses than in typical therapeutic regimens (e.g., 4 mg 2–3x/day) without observed toxicity. Limitations were the inability to perform standard FEV1 assessments due to advanced patient age and comorbidities. Prior long-term steroid use complicated monitoring of corticosteroid-related adverse effects. Despite these limitations, the study indicated that eDSP was safe in elderly COPD patients with comorbidities, and it reduced the need for other systemic or inhaled therapy.

## 3 eDSP in cystic fibrosis

This study included 17 (12–26 years old) patients with CF. Patients 1–9 received a single or repeated infusions of eDSP to assess safety, reproducibility of drug loading into RBC, and PK. The dose ranged from 1.19 mg to 14.5 mg of eDSP. Doses were increased by adjusting erythrocyte incubation times during encapsulation (5–30 min). Patients 9-17 (one patient was included in both groups) received an eDSP infusion every 4 weeks for 15 months to evaluate clinical efficacy. Nine controls were treated with standard of care approaches for CF. The average dose in the efficacy cohort was 8.9 ± 3.8 mg, with approximately 16 infusions per patient (Rossi et al., 2004).

Theapproach was well tolerated, with no observable toxicity or adverse events related to loading procedures. PK analysis confirmed maintenance of therapeutic plasma levels of dexamethasone for up to 28 days post-infusion. Increased dose of eDSP resulted in increased plasma dexamethasone concentrations, supporting a once-monthly dosing schedule. Over 15 months of treatment, patients showed statistically significant improvement in lung function (mean FEV1 increased from 57% ± 14% to 61% ± 19% of predicted, p = 0.01) and a 51% reduction in relapses of *Pseudomonas aeruginosa*-related infections. Matched control patients experienced a decline in FEV1 and a 22% increase in infection rate. Additionally, plasma interleukin-8 levels decreased over time in treated patients, reflecting reduced systemic inflammation. Patients reported improved quality of life.

Key benefits of eDSP included consistent drug release, improvement in clinical outcomes using very low glucocorticoid doses (10x lower than standard oral regimens), reduced hospitalization frequency, and improved quality of life. Limitations included inter-patient variability in RBC drug loading, the need for specialized equipment and handling to produce eDSP, the small study size, lack of a placebo group, and relatively short follow-up of treated patients. Nonetheless, the use of erythrocytes to deliver corticosteroids was considered a promising strategy to achieve anti-inflammatory benefits in chronic diseases like CF while minimizing systemic corticosteroid toxicity.

## 4 eDSP in cystic fibrosis–continuation of previous study

This study was an open label continuation of Rossi et al., 2004. Out of 17 patients enrolled in Rossi’s trial, 9 continued to receive monthly treatments for the full 24 months, while 8 patients received less than 4 treatments and discontinued treatment due to personal choice or perceived logistical difficulties. The experimental treatment involved monthly infusions of eDSP, delivering sustained plasma levels of dexamethasone of ∼0.1–0.2 nmol/mL over 1 month. Nine matched controls received standard therapy (Lucidi et al., 2006).

eDSP therapy was well tolerated, and none of the patients in the experimental group developed diabetes, cataracts, hypertension, striae, increased intraocular pressure or decrease in BMD. Over 24 months, FEV1values improved in the eDSP group from an average of 53.8%–59.8%, whereas the control group experienced a decline from 56.8% to 53.2% (p = 0.04 for the difference in FEV1 Z-score trends). The number of infectious exacerbations was slightly higher in the eDSP group, possibly reflecting increased monitoring of the treatment group as opposed to the control group. It was estimated that a larger study would be needed for a more definitive trial.

## 5 eDSP in steroid-dependent mixed Inflammatory bowel disease

Table 2 summarized studies of eDSP in inflammatory bowel disorders.

**TABLE 2:** Summary of eDSP studies in Crohn’s disease and ulcerative colitis.

Study parameter	Annese et al. (2005) Adult Crohn’s Disease and UC Pilot uncontrolled study	Castro et al. (2007) Pediatric Crohn’s Disease Pilot uncontrolled study	Bossa et al. (2008) Adult UC Phase II randomized, controlled trial	Bossa et al. (2013) Adult steroid-dependent UC Randomized, placebo-controlled, double-blind study
Indication	Steroid-dependent inflammatory bowel disease (Crohn’s and UC)	Pediatric patients with steroid-dependent Crohn’s disease	Adults with mild to moderate UC, refractory to mesalamine	Adults with steroid-dependent ulcerative colitis
Treatment groups	5 patients with Crohn’s disease, 5 with UC, no controls	18 patients, no controls	20 eDSP patients, 10 patients on oral prednisolone (0.5 mg/kg), 10 patients on placebo infusions	19 eDSP patients, 19 placebo controls
Patient age	Mean age 39 ± 14 years for UC patients and 33 ± 6 years for Crohn’s disease patients Range 28-62 years	Mean age 13.7 years Range 5.4 to 18 years	Mean age 46.8 ± 13.7 years Range 20-69 years	Median age 35 years (IQR 26-47) for eDSP group Median age 27 years (IQR 22-35) for placebo group
Dose of eDSP	Mean 5.5 mg ± 2.4 mg per infusion (range 1-10.9 mg)	Mean 8.8 mg per infusion (range 2.4-14.7 mg)	Mean 9.9 mg ± 4.1 mg per infusion	Mean 9.8 ± 4.6 mg per infusion for eDSP group
PK findings	Biphasic decline in plasma dexamethasone concentration described	No PK other than eDSP levels	Biphasic decline in plasma dexamethasone concentrations, stable levels at day 14	After the initial peak, dexamethasone concentrations declined, reaching a steady-state concentration of 8-10 ng/mL which lasted for 28 days
Frequency of treatment	Every 4 weeks x 3	Every 4 weeks for 24 months	Every 2 weeks x 2	Every month x 6
Outcome measures	Clinical and endoscopic remission (CDAI/DAI), CRP and ESR, steroid withdrawal	pCDAI, endoscopy, histology, cortisol levels, BMI	Clinical remission, endoscopic remission, decline in CRP and ESR	Steroid-free remission, CRP, mucosal healing
Time of assessment	16 weeks after the start of treatment follow up at 12 months	24 months after the start of treatment	8 weeks after the start of treatment	6 months after the start of treatment
Treatment outcomes	After 2 months all patients stopped oral steroids CRP and ESR declined. At 12 months 60% of patients relapsed	78% of patients discontinued oral corticosteroids, 44% had endoscopic remission, pCDAI declined significantly, hospitalizations were reduced	Clinical remission 75% in eDSP group, 80% in oral corticosteroid group, and 10% in placebo control. CRP and ESR reduced in eDSP and oral corticosteroid groups	13/19 (68%) treated patients and 4/18 (22%) placebo controls were off steroids and stable at 6 months (p = 0.008). 4/19 treated patients and 1/18 placebo controls had mucosal healing (NS). 12/13 treated patients and 3/4 placebo patients subsequently relapsed
Steroid-related adverse events	None	No serious adverse events, transient cortisol suppression on the day after the infusion, returning to normal within 3 days, BMI stable, BMD improved in 33% of participants	No steroid-related adverse events in eDSP group, 8/10 patients in oral corticosteroid group developed steroid-related adverse events	Steroid-related adverse events we present in 74% of eDSP patients at baseline and in 26% at month 6. In the placebo group, 72% of patients had steroid-related adverse events at both assessments
Main findings of the study	Very low doses of eDSP were able to maintain IBD patients in clinical remission and allowed oral corticosteroids taper	Confirmed that the morning cortisol suppression was limited to less than 3 days after the eDSP infusion. Confirmed absence of adverse effects on BMD in children	eDSP was as effective as oral corticosteroids in treatment of UC but at a significantly lower dose and with fewer side effects	eDSP infusion enabled steroid withdrawal in most patients during therapy with significant reduction in steroid-related adverse events. Maintenance therapy is needed to sustain the response

BMI, body mass index; BMD, bone mineral density; CDAI and pCDAI , Crohn’s disease activity index and pediatric Crohn’s disease activity index; CRP, C-reactive protein; DAI, disease activity index; eDSP, encapsulated dexamethasone sodium phosphate; ESR, erythrocyte sedimentation rate; IBD, inflammatory bowel disease; IQR, interquartile range; NS, not statistically significant; PK, pharmacokinetics; IL-8, interleukin 8; TNF-α, tumor necrosis factor alpha; UC, ulcerative colitis.

In this open-label pilot trial Annese et al., 2005, monthly eDSP infusions were given to adult patients (28–62 years of age) with steroid-dependent IBD (5 with CD and 5 with UC). At baseline, patients were on daily prednisone ranging from 8 to 24 mg. The mean dose of eDSP was 5.5 ± 2.4 mg per infusion given every 4 weeks for a total of three infusions. Patients were followed for 12 months. Outcomes were measured at 16 weeks and included clinical and endoscopic remission and serological markers of inflammation C-reactive protein (CRP) and erythrocyte sedimentation rate (ESR). At week 16, all patients were in clinical remission with statistically significant declines in ESR and CRP. Endoscopically, 3 patients were in complete remission, and 7 had evidence of disease persistence. All patients were able to discontinue oral corticosteroids after 2 months of eDSP treatment and stay off steroids for a mean of 6 months after the first eDSP infusion. At 12-month, 6 patients had relapsed and 4 remained in remission. No new steroid-related adverse events were noted, and pre-existing steroid-related adverse events had improved at follow-up.

Limitations included the lack of a placebo group, considering the 30%–40% effectiveness of placebo in maintenance trials of IBD. Variability in DSP encapsulation, explained by patients’ erythrocyte characteristics and variability in procedures, was also noted.

## 6 eDSP in Pediatric Crohn’s disease-a case report

A 16-year-old girl with a 6-year history of ileo-colic CD with numerous relapses, pelvic abscess and rectovesical and enterocutaneous fistulas, previously treated with mesalamine, prednisone, budesonide, azathioprine, infliximab and elemental nutrition, was enrolled in a single-patient trial. She started monthly eDSP treatment (dose not provided), and after 4 infusions both fistulas had closed. After 24 months of treatment with eDSP, she showed endoscopic and histological remission. In addition to eDSP, she continued treatment with mesalamine and azathioprine. At the time of publication, she was on eDSP for 3 years and during treatment never presented with steroid-related side effects such as high blood pressure or Cushingoid features. Her BMD, which was −1.3 z-scores at the time of start of eDSP, did not worsen (Castro et al., 2006).

The authors concluded that eDSP was safe and effective in a patient with fistulizing CD which did not respond to conventional treatments.

## 7 eDSP in Pediatric Crohn’s disease

This study included 18 pediatric steroid-dependent CD patients (mean age 13.7 years). Steroid dependency was defined as a relapse during steroid tapering or shortly after discontinuation of steroids. At the start of eDSP treatments, patients were on ≥10 mg of oral prednisone. A mean eDSP dose of 8.8 mg (range 2.4–14.7 mg) was infused every 4 weeks for 24 months. Endpoints included Pediatric Crohn’s Disease Activity Index (pCDAI), BMD by dual-energy X-ray absorptiometry, body mass index, morning cortisol levels, and steroid dose reduction (Castro et al., 2007).

After 24 months, pCDAI scores decreased from 23.8 to 9.6 (p < 0.05), indicating clinical improvement. Fourteen patients (78%) were able to discontinue oral corticosteroids, while the rest reduced their doses significantly. Endoscopic remission was observed in 44% of patients. Despite favorable patient-reported outcomes and the reduction in pCDAI, 10 patients did not show improvement in histological findings. BMD improved in 33% of participants and remained stable in the rest. Morning cortisol levels were transiently suppressed the day after the infusion, followed by fast normalization of levels within 3 days after eDSP. No significant adverse effects or changes in blood pressure or heart rate were observed. Additionally, hospitalization rates were reduced by more than 50% during eDSP treatment.

The authors concluded that eDSP was safe and effective in patients with steroid-dependent, mild and moderately active CD, and recommended confirmatory studies.

## 8 eDSP in adults with ulcerative colitis refractory to mesalamine

This was a phase 2 randomized controlled trial of eDSP in 40 patients with mild-to-moderate UC, refractory to mesalamine after 2 months of treatment. Patients were divided into 3 groups: A) 20 patients received 2 eDSP infusions 14 days apart, mean eDSP dose of 9.9 ± 4.1 mg; B) 10 patients were treated with oral prednisolone 0.5 mg/kg for 14 days with 6 mg/week taper subsequently; C) 10 patients received placebo infusions. All patients remained on oral and topical mesalamine. Patients in group A were older, had a longer history of disease (mean of 108 ± 76 months), and 80% previously used standard corticosteroids. Patients were assessed at baseline and at 8 weeks for clinical and endoscopic remission, inflammatory markers (CRP and ESR), cortisol levels, and adverse events (Bossa et al., 2008).

Clinical and endoscopic remission at 8 weeks was achieved in 75% (15/20) of the eDSP group, 80% (8/10) of the oral prednisolone group, and 10% (1/10) of the placebo group. These improvements in remission rates with eDSP and oral prednisolone were statistically significant (p < 0.001) compared with placebo. Mean values for ESR and CRP were also improved in groups A and B. Patients in clinical remission at week 8 were followed for time to relapse. The relapse occurred after a mean of 12 ± 5 months in the eDSP group (n = 15), 14 ± 6 months in the oral prednisone group (n = 8), and at 12 months in the placebo group (n = 1). The mean total received steroid dose was 18.4 ± 7.4 mg of eDSP in group A and 870 ± 120 mg of prednisolone in group B.

PK findings indicated a biphasic decline in plasma dexamethasone concentration. The peak value was found after the eDSP infusion at time 0, reflecting dephosphorylation by erythrocyte enzymes. In the second phase, the levels were stable and detectable for 14 days after the infusion.

While there were no steroid-related adverse events reported in eDSP and placebo patients, patients receiving oral prednisolone developed typical steroid-related side effects including acne (8/10), hirsutism (2/10), weight gain (5/10), amenorrhea (2/4), and insomnia (1/10). At 8 weeks, 1/10 patients in the eDSP group and 6/10 patients in the prednisolone group had abnormally low cortisol level. The authors concluded that eDSP was effective at a significantly lower doses than oral prednisolone and had fewer side effects.

## 9 eDSP in adults with steroid-dependent ulcerative colitis

This was a phase 2, randomized, double-blind, placebo-controlled study of eDSP in 37 patients with steroid-dependent UC (requiring ≥10 mg of prednisone daily for at least 3 months and unable to reduce the dose due to recurrence of disease). Nineteen patients received eDSP and 18 received a placebo, for a total of 6 monthly infusions. At study entry, patients were on a median dose of 20 mg of oral corticosteroids daily (range 10–45 mg) (Bossa et al., 2013).

The mean monthly eDSP dose, measured in the first 10 patients, was 9.8 ± 4.6 mg. PK indicated biphasic elimination of dexamethasone with steady plasma levels after the initial peak at 24 h, and persistence of dexamethasone for 28 days. The primary outcome was the proportion of patients discontinuing oral corticosteroids while maintaining clinical remission or stable disease. Steroid tapering started after the second infusion. Steroid-free remission/stable disease was achieved in 68% (13/19) of patients receiving eDSP and in 22% (4/18) of placebo patients (p = 0.008). Two patients receiving eDSP and 11 patients receiving placebo discontinued treatment early due to deterioration of disease upon oral corticosteroid taper. Endoscopic mucosal healing was documented in 4 (21%) patients receiving eDSP and in 1 (5%) patient receiving placebo.

Among patients who completed the 6 planned treatments, 13/19 (68%) patients in the eDSP group and 4/18 (22%) in placebo group were able to discontinue oral corticosteroids and were observed on mesalamine and/or other immunosuppressants but off corticosteroids. Fifteen of them (88%) relapsed (all but one in each group, respectively) at a mean of 2.4 ± 1.4 months, irrespective of treatment assignment.

Steroid-related side effects were documented at baseline in 74% of eDSP patients and in 72% of placebo patients. At the end of treatment, steroid-related adverse events were documented in 26% of eDSP patients and 72% of placebo patients (p = 0.008).

The authors concluded that monthly eDSP infusions enabled steroid withdrawal in most patients without relapse during therapy, and the treatment resulted in a significant reduction of steroid-related side effects. The occurrence of relapses following discontinuation of eDSP indicated the need for maintenance therapy in this group of patients. A limitation of this study is that it was conducted over a long period (2003–2012) in a single institution. Emerging new biological treatments for UC also hampered patient enrollment.

## 10 Conclusion

Early clinical trials of eDSP demonstrated that treatment was feasible and could be used in children as well as older adults. PK analyses demonstrated dexamethasone levels up to 28 days post-infusion. Notably, the mean dose used in these early trials did not exceed 10 mg of eDSP per infusion suggesting that glucocorticoid receptor occupation could be achieved with a low eDSP dose. The absence of expected steroid-related side-effects across studies was remarkable.

These early trials had small sample sizes, lack of multicenter validation, and inconsistent control groups hampering efficacy confirmation. Observed technical challenges and variability in erythrocyte loading prompted continued improvement of the technology and process controls, leading to automation and reliable drug loading into erythrocytes.

Due to systemic toxicities, the use of corticosteroids in inflammatory disorders continues to decrease since the early 2000s and has been supplemented by biologic agents. Biologics target specific cytokines or receptors, which limit their efficacy to the subgroup of patients with a disease driven by that pathway. Corticosteroids, through their global suppression of inflammatory gene expression across multiple pathways, lead to reliable and rapid symptom control in a wide range of patients. Delivery of eDSP minimizing systemic exposure and toxicities, described in these early clinical studies and subsequently confirmed in children with ataxia telangiectasia treated for longer than 2 years, warrants a renewed attention to use of corticosteroids delivered via RBC in selected inflammatory conditions.
